# Detection Line Spectrum of Ship Radiated Noise Based on a New 3D Chaotic System

**DOI:** 10.3390/s21051610

**Published:** 2021-02-25

**Authors:** Gang Hu, Kejun Wang, Liangliang Liu

**Affiliations:** 1 College of Automation, Harbin Engineering University, Harbin 150001, China; hugang@hrbeu.edu.cn (G.H.); liuliangliang@hrbeu.edu.cn (L.L.); 2 School of Mathematics and Information Sciences, Anshan Normal University, Anshan 114007, China

**Keywords:** chaos system, SE and *C*_0_ complexity, weak signals, circuit implementation

## Abstract

This paper proposes a new detection model of a weak signal based on a third-order chaotic system. Using a dynamic analysis tool, such as the Lyapunov exponent and the bifurcation diagram, variations of dynamic behavior can be observed, and the weak signal underwater can be picked up. In order to improve the observability of detection signals in the time domain and frequency domain, the spectral entropy complexity algorithm (SE) and *C*_0_ complexity algorithms are used to analyze and extract the weak signal. The experimental results show that the spectrum extraction based on the complexity algorithm can accurately reflect the dynamic characteristics of the detected signal. It provides the theoretical direction and experimental data support for the application of the chaotic system in the field of acoustic detection.

## 1. Introduction

Line spectrum features are one of the important features of ship-radiated noise [1], and the sound sources of ship line spectrum features are mainly from a ship’s mechanical noise [1,2] and propeller noise [3,4]. In the process of sailing, the vibration of the rotating machinery of the ship power system and the rotation of propeller blades will inevitably radiate periodic noise to the surrounding environment [5,6,7,8]. Moreover, the characteristics of a ship’s line spectrum are stable and unique [9]. In the task of passive sonar target detection and recognition [10,11,12], the detection and extraction technology of a ship’s line spectrum plays a key role [13]. Therefore, in passive sonar signal processing, the detection and extraction of a ship’s line spectrum features have always been a hot research issue in the field of underwater acoustics; it has important application value in the field of national defense. There are many traditional methods to detect and extract a ship’s line spectral features, which are mainly based on spectral analysis and random systems. Due to the complexity of ocean background noise, when the ship is far away, or the signal is very weak, the traditional weak-signal detection method can only detect the weak signal of SNR(Signal-to-Noise Ratio) above −10 dB [14,15]. It is very difficult to detect and extract the characteristics of a ship’s line spectrum, so it is limited in practical application. In recent years, with the emergence of ship stealth technology, the signal-to-noise ratio of ship noise in passive sonar signals has been greatly reduced. Under the condition of a low signal-to-noise ratio, the detection and extraction of a ship’s line spectral features is faced with great challenges and problems [16].

Weak signal detection technology is the science of signal detection, which has developed vigorously in the past decades. It is widely used in physics, biology, artificial intelligence and in other fields. However, due to their lower energy, weak signals are difficult to be recognized and detected effectively under the influence of a strong noise environment. Moreover, due to the limited accuracy of sensors and other measuring tools, many weak signals cannot be effectively detected and extracted. With the continuous progress of signal detection technology, detection equipment and instruments are constantly updated. In 1962, the first amplifier for weak signal detection was invented by PARC [17], which announced the industrialization of weak-signal detection technology. Then, Klein invented the Boxcar integrator and successfully realized the electronic sampling integral [18]. Weak-signal detection has always been a difficult problem in signal processing, and it is also one of the top subjects in the field of measurement and control research. It is related to the progress and development of the humanities and social sciences and is closely related to the production and life of human beings. Therefore, the research on weak-signal detection technology is of great value to promote the progress of various scientific fields.

Because a chaotic oscillator is sensitive to weak signals of the same frequency, it can effectively resist a large number of noisy signals. The chaotic oscillator has an obvious advantage in weak periodic signal detection. Especially, it can detect extremely weak periodic signals. Recent studies have shown that, based on the sensitivity of the initial state and good anti-noise ability, weak signals can be effectively analyzed and extracted by the chaos oscillator. BIRX team used the principle of chaotic resonance to separate weak signals from strong noises, and the line spectrum of weak chaos can be effectively extracted [19]. Short et al. proposed the weak chaotic encryption signal from the communication system by using the long-term boundedness principle of chaotic signals [20,21]. Wang proposed a weak signal detection method under a white noise environment through Duffing moment detection [14,15,22]. Li et al. conducted a preliminary study on the line spectrum components of underwater target signals, and the results showed that chaotic oscillators could effectively detect the weak line spectrum with known frequencies. Because of the limitation of the existing system, the research progress of detection based on the chaos model is slow. Thus, it is necessary to propose a new detection model for underwater acoustic detection. Especially, some weak signals are too weak in energy, and they cannot be effectively extracted from the line spectrum [23,24,25,26,27]. Based on the above potential problems in weak signal underwater acoustic detection, a new chaos system model that can be applied in the detection of the chaotic weak signal is proposed in this paper. In comparison with the common dynamic analysis method, such as the Lyapunov exponent and the bifurcation diagram, spectral entropy (SE) and *C*_0_ complexity as another dynamic analysis tool is used. Additionally, they can effectively and accurately reflect the influence of weak signal and noise signal on the energy value of the chaos sequence. It provides a new analysis method of engineering and theoretical guidance for research in related fields.

In this paper, a new chaotic system model for detecting underwater weak signals is proposed. SE and *C*_0_ complexity [20] algorithms are used to analyze the weak signals, and the complexity characteristic curves of the detected signals in the strong noise environment are extracted. The Lyapunov exponent and the bifurcation diagram, combined with SE and *C*_0_ complexity curves, were used for numerical simulation analysis, and the proposed detection system was implemented by circuit simulation. The experimental results show that the proposed new chaotic system model is feasible for underwater weak signal detection, and the proposed complexity algorithm can effectively analyze and extract weak signals in different frequency states.

## 2. System Model

### 2.1. The Proposed System Model

The proposed system model, which can be applied in the weak signal detection, is a third-order chaotic system model based on the system structure of Sprott system [28], and its system model can be expressed as:

(1) $$\left\{\begin{array}{c} \dot{x}=y-x\\ \dot{y}=axz\\ \dot{z}=b-xy \end{array}\right.$$

**Figure 1:**
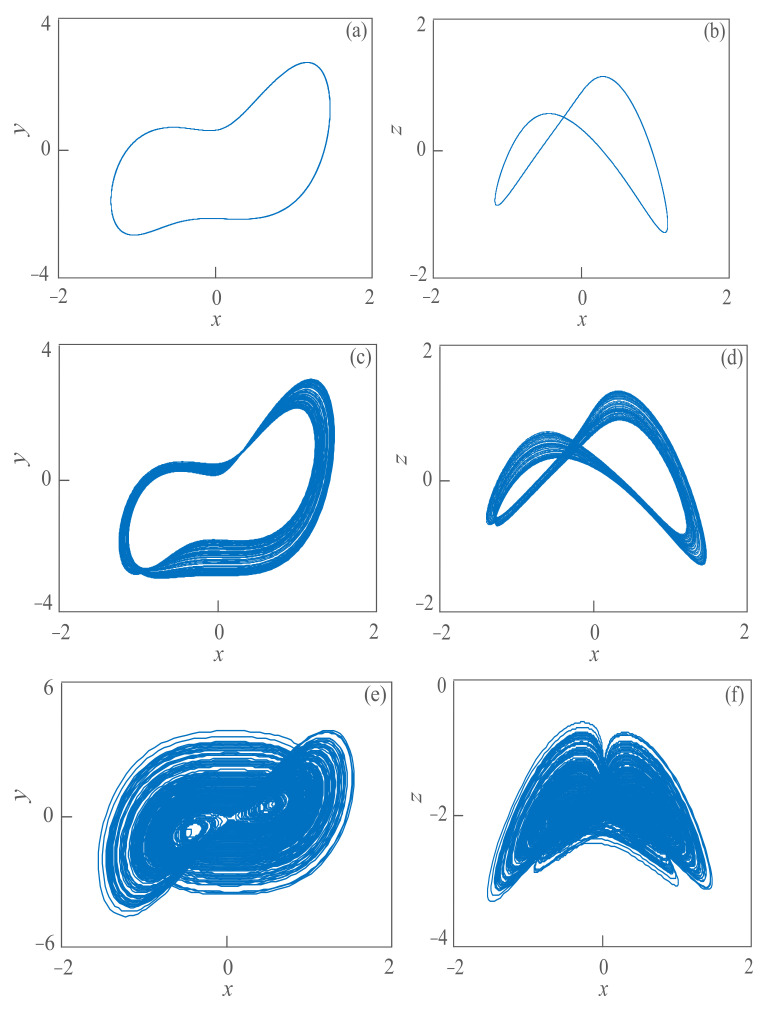
The attractor phase diagram of the proposed system. (**a**) The attractor of periodic state on *x*−*y* plane; (**b**) the attractor of periodic state on *x*−*z* plane; (**c**) the attractor of weak chaos state on *x*−*y* plane; (**d**) the attractor of weak chaos state on *x*−*z* plane; (**e**) the attractor of chaos state on *x*−*y* plane; (**f**) the attractor of chaos state on *x*−*y* plane.

In the above formula, *x*, *y* and *z* are the state variables of the system, *a* and *b* are the parameter variables of the system; the initial state is (0.1, 0.1, 0.1) and the time step is 0.01 s. When *a* = 12 and *b* = 0.5, the periodic attractors of the system can be observed, as shown in Figure 1a,b. When *a* = 12 and *b* = 0.4, the weakly chaotic attractor of the system can be observed, as shown in Figure 1c,d. When *a* = 12 and *b* = 0.3, the chaotic attractors of the system can be observed as shown in Figure 1e,f.

### 2.2. Weak-Signal Detection

When the system detects a weak signal, the results between existing theory and practical application are different. The reason is that the assumed signal is usually a signal with a specific frequency and white Gaussian noise mixture, so it also contains a variety of frequency components, and most of them contain periodic interference signals. Since *A*_1_cos(*w*_1_*t*) is the useful frequency signal to be measured, and *A*_2_cos(*w*_2_*t*) is a noise signal of a certain frequency that is mixed with period and noise interference, so the mathematical model, after introducing the weak signals and noisy signals, can be express as:

(2) $$\left\{\begin{array}{c} \dot{x}=y-x\\ \dot{y}=axz+A_{1}\cos\left(w_{1}t\right)+A_{2}\cos\left(w_{2}t+\varphi \right)\\ \dot{z}=b-xy \end{array}\right.$$

**Figure 2:**
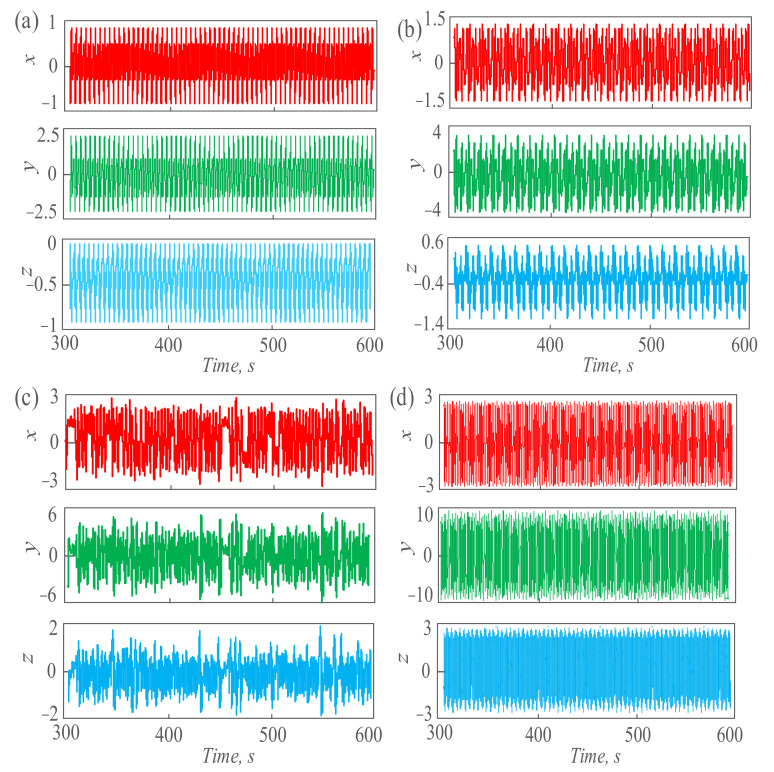
The chaos sequence is generated by the proposed system under different dynamic behavior. (**a**) The chaotic sequence in period−1; (**b**) the chaotic sequence in period−3; (**c**) the chaotic sequence in a chaotic state; (**d**) the chaotic sequences in large−scale chaotic states.

In the above formula, *x*, *y*, *z* are the state variables of the system, *a*, *b* are the parameter variables of the system, the initial value is (0.1, 0.1, 0.1), and the time step is 0.01 s. *A*_1_ is the amplitude of the weak signal, *A*_2_ is the amplitude of the noise signal, *w*_1_ is the angular frequency of the weak signal, *w*_2_ is the angular frequency of the noise signal, and *φ* is the initial phase of the noise signal. With the change of each parameter, the model can produce different dynamic behaviors. When *A*_1_ = 1, *A*_2_ = 2, *w*_1_ = 1, *w*_2_ = 2, *φ* = 0, *a* = 12 and *b* = 0.2, as shown in Figure 2a, we can observe the chaotic sequence of the system containing the detection signal and noise signal within 200~600 s. When *A*_1_ = 1, *A*_2_ = 2, *w*_1_ = 1, *w*_2_ = 2, *φ* = 0, *a* = 12 and *b* = 0.5, the chaotic sequence of the system containing the detection signal and noise signal is as shown in Figure 2b. When *A*_1_ = 1, *A*_2_ = 2, *w*_1_ = 1, *w*_2_ = 2, *φ* = 0, *a* = 12 and *b* = 0.9, the chaotic state sequence of the system is as shown in Figure 2c. With other parameters unchanged and set *b* = 3, the chaotic sequence of the system in the large-scale chaotic state is shown in Figure 2d. When the state of the system is transferred, the dynamic state will also change significantly. Based on these changes, we can judge and detect the existence of weak signals and make an effective extraction.

## 3. Weak Signal Detection and Line Spectrum Extraction Based on a Complexity Algorithm

### 3.1. The Description of SE Complexity Algorithm

The spectral entropy (SE) complexity algorithm can accurately show the complexity of the system. The higher value of complexity shows the stronger chaotic behavior generated by the chaos system. It can be described:

(3) $$\tilde{x}(n)=x(n)-1/n\overset{N}{\underset{i=1}{\sum }}x(i)$$

here, *x*(*n*) is the amplitude of the sequence. Then, do computing the discrete Fourier transform, we can get:

(4) $$X(k)=\overset{N-1}{\underset{n=0}{\sum }}\tilde{x}(n)e^{-j\frac{2\pi }{N}nk}$$

where, *k* = 0, 1, 2, …, *N* − 1. Then, we can calculate the relative power spectrum:

(5) $$q\left(k\right)=\frac{1}{N}|X\left(k\right){|}^{2}$$

in Equation (5), *k* = 0, 1, 2, …, *N*/2 − 1, and we can get the total power:

(6) $$\tilde{q}=\overset{N/2-1}{\underset{k=0}{\sum }}|X\left(k\right){|}^{2}$$

So the probability of power spectrum is:

(7) $${\tilde{q}}_{k}=\frac{q\left(k\right)}{q}=\frac{\frac{1}{N}|X\left(k\right){|}^{2}}{\frac{1}{N}\sum_{k=0}^{N/2-1}|X\left(k\right){|}^{2}}=\frac{|X\left(k\right){|}^{2}}{\sum_{k=0}^{N/2-1}|X\left(k\right){|}^{2}}$$

Combined with the above formula, we can obtain the SE complexity:

(8) $$SE=-\ln(N/2)\overset{N/2-1}{\underset{k=0}{\sum }}q_{k}\ln{\tilde{q}}_{k}$$

*C*_0_ complexity algorithm is a complexity algorithm based on the Fourier transform. Its calculation algorithm is:

(9) $$X(k)=\overset{N-1}{\underset{n=0}{\sum }}x(n)e^{-\frac{2\pi }{N}nk}$$

in Equation (9), *k* = 0, 1, …, *N* − 1, *X*(*k*) is the sequence after Fourier transform. Then, introducing tolerance parameter *c*, reserve the mean square value of more than *c*, we have:

(10) $$\hat{X}\left(k\right)=\left\{\begin{array}{cc} \hat{X}\left(k\right), & |X\left(k\right){|}^{2}>cX\left(k\right)\\ 0, & |X\left(k\right){|}^{2}<cX\left(k\right) \end{array}\right.$$

Then, with an inverse FFT for Equation (10), we have:

(11) $$\tilde{x}(n)=1/N\overset{N-1}{\underset{k=0}{\sum }}\hat{X}\left(k\right)e^{j2\pi nk/N}$$

in which *n* = 0, 1, …, *N* − 1. According to the above equations, *C*_0_ complexity can be obtained:

(12) $$C_{0}(c,N)=\overset{N-1}{\underset{n=0}{\sum }}|x(n)-\tilde{x}(n)|$$

The dynamic behavior can be simulated numerically based on SE and *C*_0_ complexity algorithms.

### 3.2. Weak-Signal Detection Based on a Complexity Algorithm

**Figure 3:**
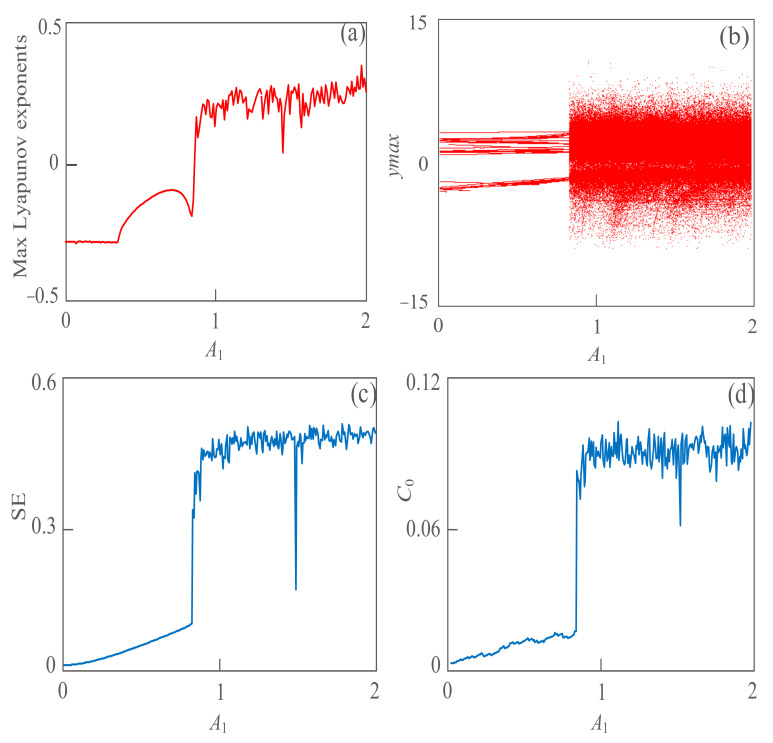
The dynamic behavior of the proposed detecting system with a different amplitude of weak signal *A*_1_. (**a**) Max Lyapunov exponent; (**b**) bifurcation diagram; (**c**) SE complexity; (**d**) *C*_0_ complexity.

A chaotic system can detect and extract weak signals in a strong noise environment because of its excellent dynamic characteristics. Small changes in the amplitude and phase of weak signals may cause changes in the dynamic behavior, and this variation can be detected by using the maximum Lyapunov exponent and bifurcation diagram, and the sensitive interval of the measured signal can be captured and extracted. Based on the new proposed detection model of a chaotic system, weak signals are detected. Set *A*_2_ = 2, *w*_1_ = 1, *w*_2_ = 2, *φ* = 0, *a* = 12, *b* = 0.2, and *A*_1_ is the change parameter. With the amplitude *A*_1_ of the signal to be tested, changes in the interval within 1~2, the maximum Lyapunov exponent can be obtained as shown in Figure 3a. From the simulation results, the maximum Lyapunov exponent of the detection model presents an increasing trend in this test range. It’s less than 0 within 0~0.874, which means that the system is in a periodic state. When the amplitude varies from 0.875~2, the maximum Lyapunov exponent of the test model increases obviously, and its value oscillates in the range of 0 to 0.5. This indicates that with the change in the weak signal to be measured, the dynamic behavior of the detection model has also changed. Especially, the chaotic state is obvious in the interval 1~2, and it is suggested to capture and extract the weak signal in this interval. Figure 3b is a bifurcation diagram of the detection model changing with the amplitude of the weak signal. From the bifurcation diagram, the proposed detection system starts to carry out a period-doubling bifurcation near 0.874 until it enters the chaotic state. When the system enters the chaotic state, the sequence oscillation amplitude of the system is larger, and the larger amplitude oscillation is conducive to searching the weak signal and effectively extracting its line spectrum. Figure 3c,d show the simulation results of the detection method based on SE complexity and *C*_0_ complexity. The system is in a periodic state within the range 0~–0.874, and the complexity values are very low or even close to 0. When *A*_1_ changes within 0.875~2, the system is in a chaotic state, and the complexity value increases instantly. The SE complexity value oscillates in the range of 0.3~0.6 and the *C*_0_ complexity value in the range of 0.06~0.12. From the simulation results in Figure 3, the variation of SE and *C*_0_ complexity is basically consistent with the variation of the maximum Lyapunov exponent and bifurcation diagram. The new detection method, based on SE complexity and *C*_0_ complexity, can effectively reflect the dynamic behavior change.

### 3.3. The Line Spectrum Extraction Based on Complexity

**Figure 4:**
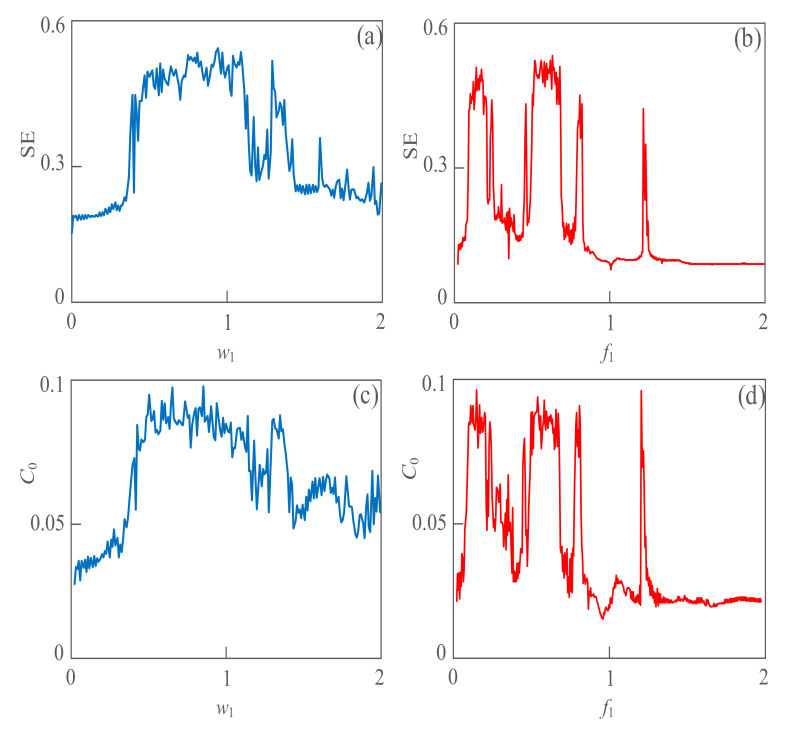

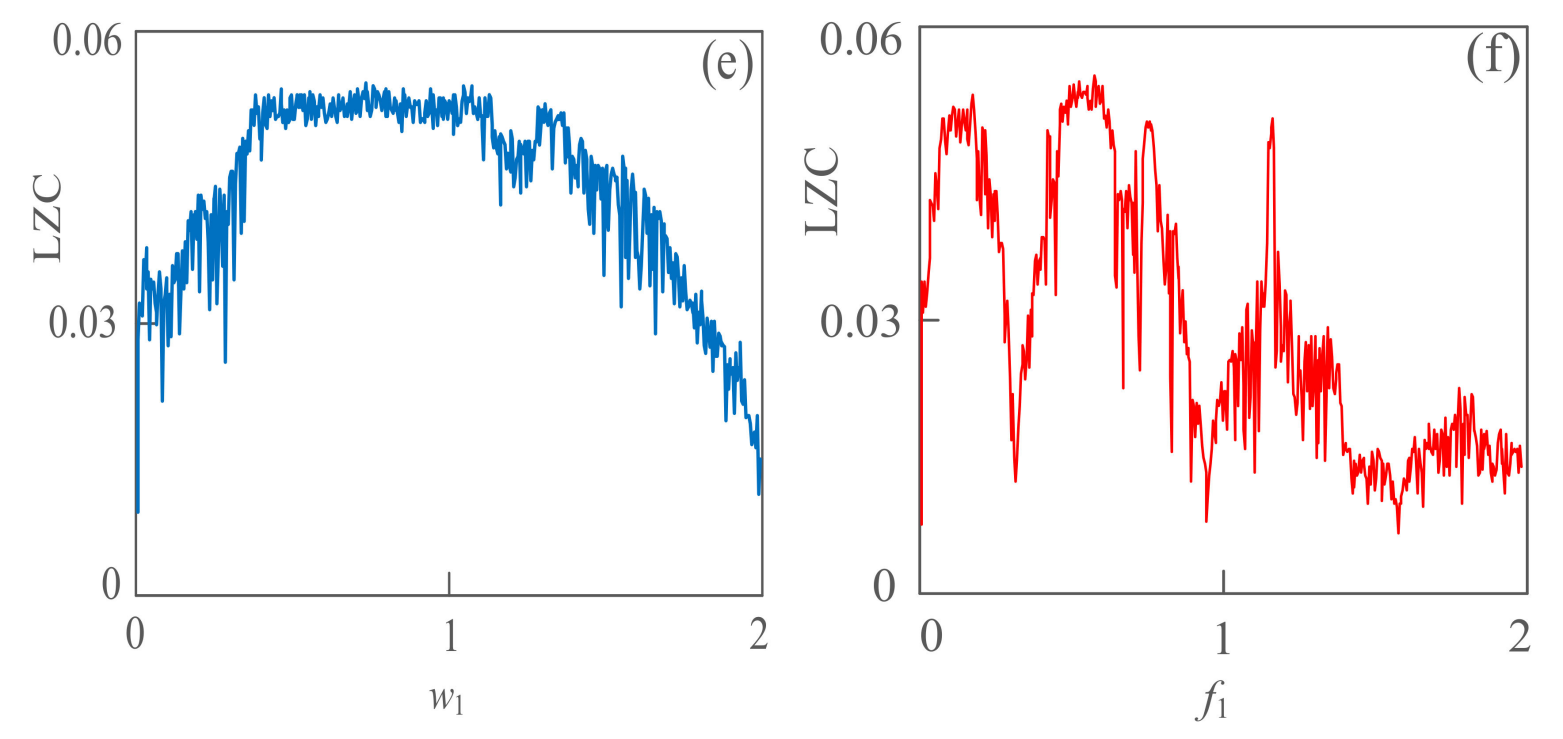
The line spectrum of the weak signal with varying frequency based on the complexity algorithm. (**a**) Line spectrum extraction based on SE complexity with a different angular frequency; (**b**) line spectrum extraction based on SE complexity with a different frequency; (**c**) line spectrum extraction based on *C*_0_ complexity with a different angular frequency; (**d**) line spectrum extraction based on *C*_0_ complexity with different frequency; (**e**) Line spectrum extraction based on LZ complexity with a different angular frequency; (**f**) line spectrum extraction based on LZ complexity with a different frequency.

**Figure 5:**
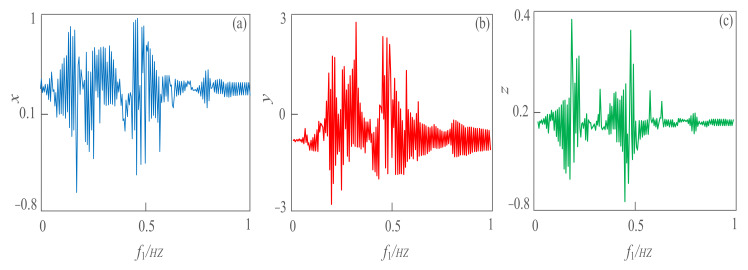
The line spectrum of the weak signal with a varying frequency. (**a**) Line spectrum extraction based on *x* direction with different frequency; (**b**) line spectrum extraction based on *y*-direction with different frequency; (**c**) line spectrum extraction based on a *z*−direction with different frequency.

From the simulation experiment in Figure 3, we can obtain that the SE complexity and *C*_0_ complexity algorithms can quantitatively detect the dynamic behavior of weak signals as well as Lyapunov exponent and bifurcation diagrams. Therefore, the signal spectrum of weak signals in a strong noise environment can be analyzed and extracted based on SE and *C*_0_ complexity algorithms. Let parameter *A*_2_ = 1, *A*_2_ = 2, *w*_2_ = 2, *φ* = 0, *a* = 12, *b* = 0.8, and the angular frequency *w*_1_ of the weak signal as the change parameter. With the change of *w*_1_ in the interval of 1~2 rad/s, SE, *C*_0_ and *LZ* (Lemple-Zie), complexity characteristic curves are shown in Figure 4a,c,e. From the simulation results, when the angular frequency *w*_1_ varies within 0.37~1.08 rad/s, the complexity value of the weak signal is larger, the maximum SE complexity value is 0.55, and the maximum *C*_0_ complexity value is 0.098. The energy of the weak signal is larger in this interval, so it is suggested to capture and extract the spectrum of the weak signal in this interval. Figure 4b,d,f show the complex line-spectrum extraction of weak signals with frequency changes, where *w*_1_ = 2π*f*_1_. With the change of *f*_1_ in the interval of 0~2 Hz, the maximum SE complexity is 0.55, and the maximum *C*_0_ complexity is 0.098. It also verifies the correctness of line spectrum extraction based on SE, *C*_0_ and *LZ* complexity algorithms. In particular, the sensitivity of the proposed system to frequency is reflected by the complexity algorithm. The line spectra of the three state variables *x*, *y* and *z* within 0~1 Hz are effectively extracted. We can predict the behavior of objects underwater that produce weak signals based on the simulation result reflected in Figure 5.

### 3.4. The Influence Analysis of Noise Signal

**Figure 6:**
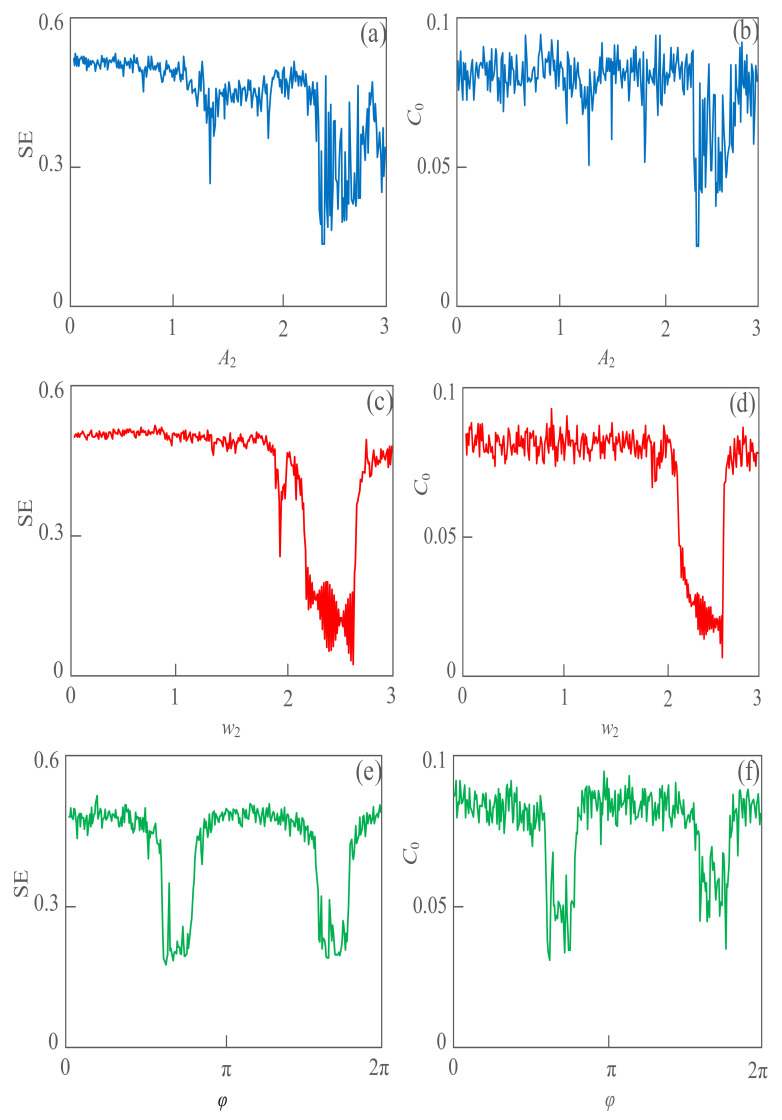
The line spectrum of the weak signal with a varying frequency, based on the complexity algorithm. (**a**) SE complexity with a different amplitude *A*_2_; (**b**) *C*_0_ complexity with a different amplitude *A*_2_; (**c**) SE complexity with different angular frequency *w*_2_; (**d**) *C*_0_ complexity with different angular frequency *w*_2_; (**e**) SE complexity with a different phase *φ*; (**f**) *C*_0_ complexity with a different phase *φ*.

In the actual detection process, the weak detection signals are often accompanied by the mixed signals of the periodic interference and Gaussian white noise. Especially, signals of the periodic interference usually contain a variety of frequency components. The detection efficiency can be effectively improved by reasonably avoiding the interference of the mixed signals of the larger energy. Select *A*_1_ = 1, *w*_1_ = 1, *w*_2_ = 2, *φ* = 0, *a* = 12, *b* = 0.8, let the amplitude *A*_2_ of the interference signal as the change parameter, and the test range is 0~3. As shown in Figure 6a,b, the complexity value of the detection system is small when the amplitude *A*_2_ changes from 2.2 to 2.6, and the minimum complexity value can be obtained in *A*_2_ = 2.24. In the detection of weak signals, it is suggested to avoid the value of interference signals in this interval. It indicates that the interference of noise signal has greatly affected the detection signal, and select *A*_1_ = 1, *A*_2_ = 2, *w*_1_ = 1, *φ* = 0, *a* = 12, *b* = 0.8, set the amplitude of the interference signal *w*_2_ as the change parameter, and the test range is 0~3 rad/s. Figure 6c,d show the influence of the interference signal on the detection signal with a different angular frequency. When the angular frequency *w*_2_ of the interference signal varies from 2.05 to 2.78, the complexity value of the detection signal is lower, and the lowest value is even close to 0.

In the actual detection process, the weak signal capture within this range should be avoided. Figure 6e,f study the interference of the initial phase of noise to the system detection signal. When the initial phase *φ* of the interference signal is fixed in the range of 0.6π~0.83π and 1.6π~1.83π, it has the greatest influence on the detection signal. A reasonable selection of the interval with less interference to the detection signal can greatly shorten the detection workload.

## 4. Circuit Implementation

Doing the circuit simulation for the proposed chaotic system, Equation (1) can be reconstructed as:

(13) $$\left\{\begin{array}{c} KRC\dot{x}=y-x\\ KRC\dot{y}=axz\\ KRC\dot{z}=b-xy \end{array}\right.$$

where *K* is the scale factor, *C* is the value of capacitance and *a* and *b* are parameters.

**Figure 7:**
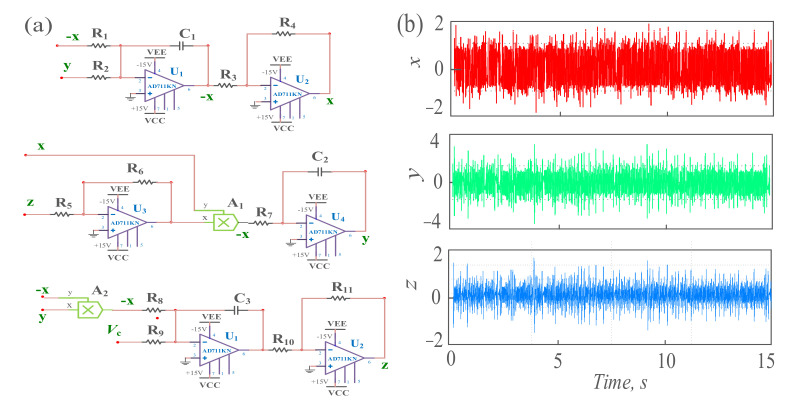
The circuit simulation of the proposed detection system. (**a**) The schematic circuit diagram; (**b**) the time-domain signal waveform diagram under the circuit simulation.

By using Equation (13), we can obtain that the schematic circuit diagram as shown in Figure 7a. Where, *C*_1_ = *C*_2_ = *C*_3_ = 10 nf, *R*_5_ = 4 kΩ, *R*_6_ = 30 kΩ, *R*_1_ = *R*_2_ = *R*_7_ = *R*_8_ = *R*_9_ = 100 kΩ, *R*_3_ = *R*_4_ = *R*_10_ = *R*_11_ = 10 kΩ, the DC power *V*c = 0.3 V and *K* = 1000. The results of circuit simulation are shown in Figure 7b. The three state variables *x*, *y* and *z* show the chaotic oscillations with different times. The results of circuit simulation are basically consistent with those of numerical simulation, the physical realizability of the proposed detection model is verified, and it can be applied to industrial actual weak signal detection.

## 5. DSP Implementation

**Figure 8:**
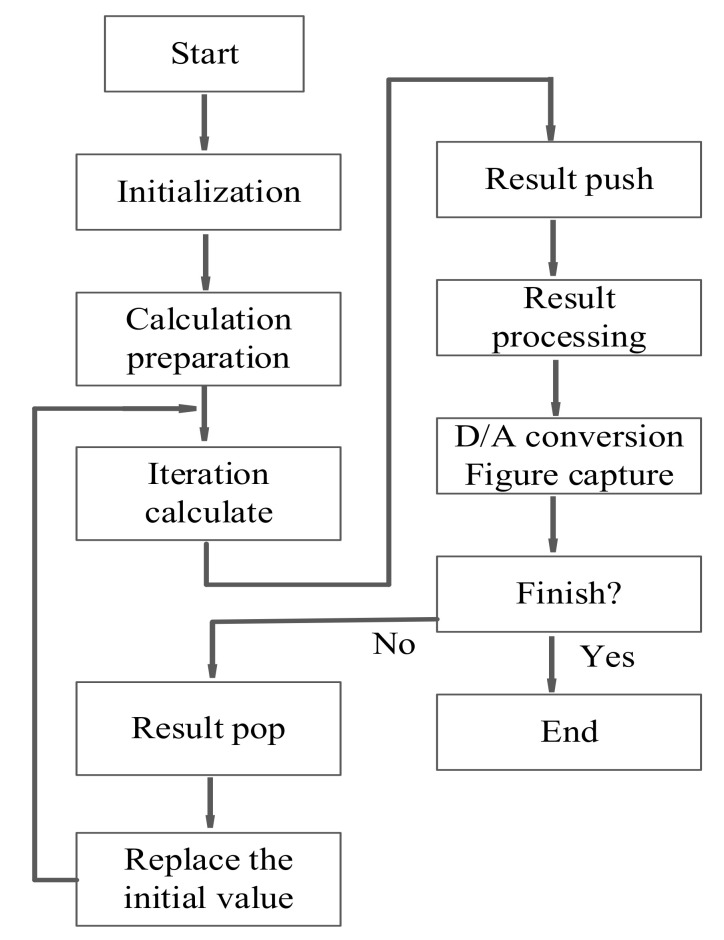
Digital signal processing (DSP) implementation flowchart.

**Figure 9:**
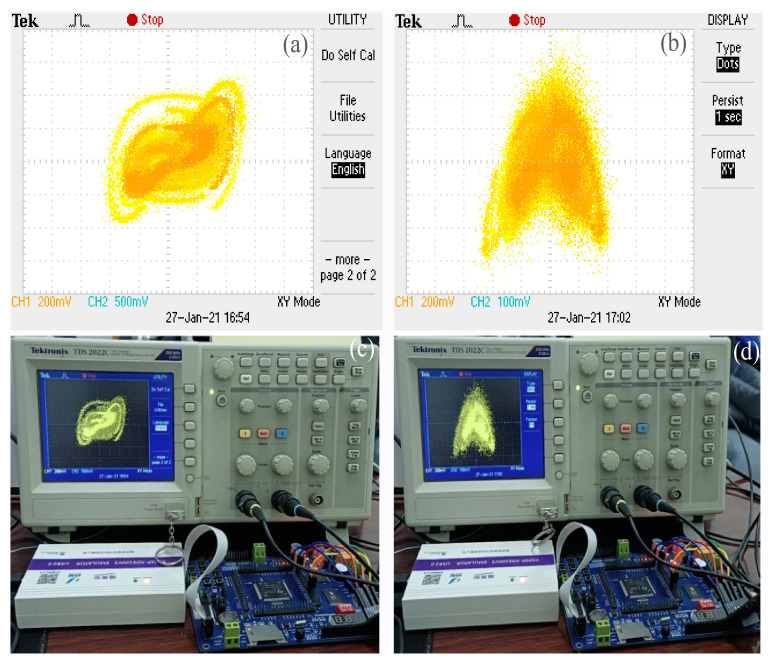
The chaotic attractors of DSP implement. (**a**) The chaotic attractor on *x*−*y* plane; (**b**) the chaotic attractor on *x*−*z* plane; (**c**) the chaotic attractor on *x*−*y* plane under laboratory; (**d**) the chaotic attractor on *x*−*z* plane under laboratory.

In order to make the proposed chaotic system better suitable for digital encryption applications, the chaotic attractors are implemented by digital signal processing (DSP) technology. During digital implementation, the dynamical degradation effect will eliminate the chaotic behavior within finite space and prevent practical applications for chaos phenomena. Set the system parameters *a* = 12, *b* = 0.3, the original state is (0.1, 0.1, 0.1), and the D/A conversion is realized by using the method of four-order Runge-Kutta. The specific implementation flowchart is reflected in Figure 8, and the chaotic attractors of DSP implement are reflected in Figure 9. The results of numerical simulation and DSP implementation are basically the same.

## 6. Conclusions

In this paper, a new chaotic system model, which can be applied to detect weak signals, is proposed. Based on the maximum Lyapunov exponent, bifurcation diagram and complexity, the weak signals can be picked up, and the line spectrum in the frequency domain can be extracted. Especially, by using the strong sensitivity of SE and *C*_0_ complexity to the chaotic behavior, the best detection interval of the weak signal can be found. The influence of the amplitude, angular frequency and initial phase of the interference signal on the detection signal is analyzed, which can effectively avoid the detection failure caused by the interference of strong noise to the detection signal; the research on the application of SE and *C*_0_ complexity algorithm in weak signal detection provides theoretical guidance and experimental verification for related fields. Next, we will continue to explore the possibility that the proposed detection model is applied in the real hydrophone, or hydro-acoustic, converter.

## Data Availability

The study did not report any data.
